# How air influences radiation dose deposition in multiwell culture plates: a Monte Carlo simulation of radiation geometry

**DOI:** 10.1093/jrr/rru022

**Published:** 2014-04-10

**Authors:** Sebastia Sabater, Roberto Berenguer, Paloma Honrubia-Gomez, Miguel Rivera, Ana Nuñez, Esther Jimenez-Jimenez, Ana Martos, Carmen Ramirez-Castillejo

**Affiliations:** 1 Department of Radiation Oncology, Complejo Hospitalario Universitario de Albacete (CHUA), C/ Hnos Falcó 37, 02006 Albacete, Spain; 2 Department of Medical Physics, Complejo Hospitalario Universitario de Albacete (CHUA), C/ Hnos Falcó 37, 02006 Albacete, Spain; 3 Centro Regional de Investigaciones Biomedicas (CRIB), Universidad de Castilla-la Mancha (UM), C/ Almansa 14, 02006 Albacete, Spain; 4 Department of Radiation Oncology, Hospital Son Espases, Carretera de Valldemossa 79, 07120 Palma de Mallorca, Spain; 5 Instituto de Salud Carlos III. Av Monforte de Lemos 5, 28029 Madrid, Spain

**Keywords:** Monte Carlo, plate irradiation, culture cells

## Abstract

Radiation of experimental culture cells on plates with various wells can cause a risk of underdosage as a result of the existence of multiple air–water interfaces. The objective of our study was to quantify this error in culture plates with multiple wells. Radiation conditions were simulated with the GAMOS code, based on the GEANT4 code, and this was compared with a simulation performed with PENELOPE and measured data. We observed a slight underdosage of ∼4% on the most superficial half of the culture medium. We believe that this underdosage does not have a significant effect on the dose received by culture cells deposited in a monolayer and adhered to the base of the wells.

## INTRODUCTION

**Fig. 1.**
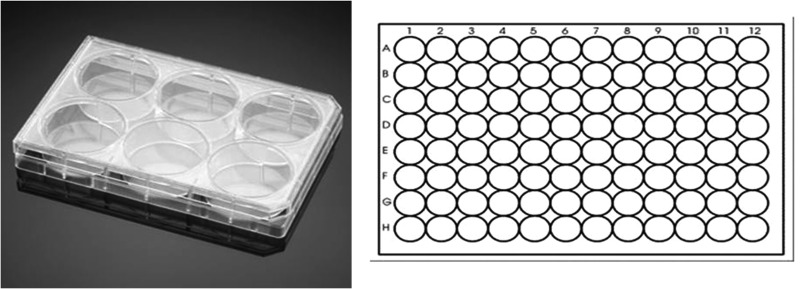
A 6-well plate photo and 96-well plate drawn showing the multiple air spaces in and around each well.

**Fig. 2.**
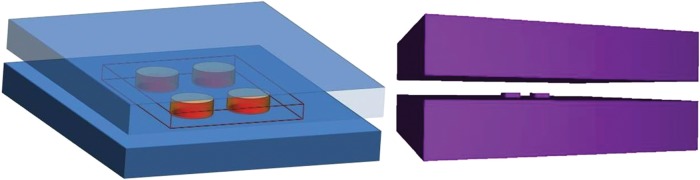
Simulated irradiation of an ideal well plate showing the water volume related to the air spaces. The drawing on the right shows the radiation geometry generated with the ‘gview3d’ software.

Irradiation is a primary tool in biological research. A great number of experimental biological studies involve and have involved culture plate irradiation. In spite of this fact, very few studies have analyzed their radiation geometry and validated the method employed. Normally when calculating the dose received to a culture plate, the total of the irradiated plate is considered to be an homogenous volume of water; in fact, cells plated in multiwell culture plates are surrounded by large amounts of air because of the existence of air cavities in such labware products. Air cavities exist in, around and within the well-plates and above the culture medium, which partially fills the well volume (Figs 1 and 2).

It is known that the presence of air cavities may produce significant underdosage as a result of the air–interfaces, depending on the energy of the photon beam, the form and size of the cavity, the size of the radiation field, and the distance from the evaluated point to the interface [1]. These underdosages are related to a lack of electronic equilibrium. This is a physical matter, but one with important issues for biologists and physicians who rely on dose deposition accuracy when planning and analyzing experimental data. To date, no information exists on how air–water interfaces influence dose deposition on culture plates.

Our aim was to quantify the grade of dose variation associated with the lack of backscatter material (build-down zone) in the zones occupied by air in the interior of the well plates.

## MATERIALS AND METHODS

The Monte Carlo (MC) code used was GAMOS v.1.9.0, which is based on the GEANT 4 code [2]. Prior to our study of the radiation geometry of cell cultures, a simple homogenous phantom 30 × 30 × 30 cm^3^ of water was prepared. This was for comparing the depth dose and profiles obtained using GAMOS with the experimental measurements performed using a silicon diode for a field size of 10 × 10 cm^2^ and 20 × 20 cm^2^ of a Siemens PRIMUS X6MV, using the phase-space provided by the IAEA for such fields [3]. (The phase-spaces chosen are computer files containing a detailed description of the particles emitted from the treatment source via an MC simulation in a defined plane.) Frequently, researchers use the files supplied by IAEA because of its creation complexity, that is also time-consuming, and in addition IAEA files have been validated versus measurements. [3]. Results were compared with another MC simulation rebuilt with the PENELOPE 2006 version. In order to be able to use the IAEA phase-spaces, a recent application of the PenEasy code (named ‘penEasy_IAEAaddon’) was employed. The irradiation geometry was generated with the ‘gview3d’ software from the PENELOPE package.

**Fig. 3.**
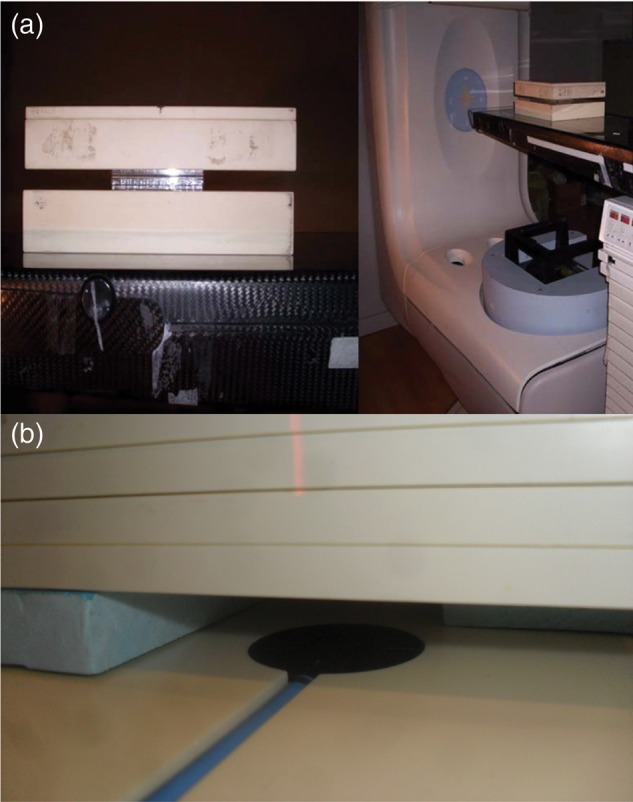
Study arrangements. (**a**) Set-up for plate irradiation. Radiation geometry of cell plates showing a posterior beam that goes through 5 cm of plastic water, a 2-cm plate culture partially filled with an aquous medium, and another 5 cm of plastic water, used as a model for the third MC simulation. (**b**) A detailed view of the arrangement for measuring underdosage with a Roos ionization chamber, used for experimental measurements and as a model for the second MC simulation.

Next, a radiation geometry like that used in cellular radiation was simulated, but minus the culture plate, i.e. 5 cm of water, 2 cm of air, and another 5-cm thickness of water. We compared the results of the simulation with the experimental measurements obtained in the zone of interest for us (the first water–air interface). In order to evaluate the underdosage, we compared the problem geometry ‘water–air–water’ (5 + 2 + 5 cm) to ‘all water’ geometry (a thickness of 12 cm). Experimental measurements were performed using X6MV photons from a Siemens PRIMUS linear accelerator at a dose-rate of 200 UM/min. Radiation was delivered via a single field at 180°, which went through 5 cm of water-equivalent material (Plastic Water^®^ from CIRS, Computerized Imaging Reference System Inc., Norfolk, VA, USA). A Ross plane-parallel ionization chamber (PTW-Freiburg, Freiburg, Germany) (Fig. 3b) was used to measure the dose in the interface between water and air. These measurements were done in order to validate the simulations results. The plane-parallel chamber is the recommended chamber for measuring percentage of depth doses (PDDs) in the build-up or build-down areas [4]. The drawback is that the chamber used has a distance of 2 mm between the electrodes. It is very important to mention that the chamber was irradiated downside. We compared the chamber response in this situation with the irradiation in the standard situation and the difference was <1%.

Finally, the goal radiation geometry was simulated with only two cubic wells (measuring 2 × 2 cm with 5 mm of thickness and 2 cm of separation) symmetrically positioned around the axis of the beam (Fig. 2). The cell-plates, which were 2 cm thick, had the wells partially filled (some 5 mm thick) and, finally, above the plates was another 5 cm of water-equivalent material. Therefore, between the surfaces of the culture medium and the water-equivalent material, there was a significant region of air (measuring 15 mm). In addition, there was air surrounding the different wells (Figs 1, 2 and 3). With this geometry, two distinct simulations were performed—with distinct voxel sizes (and tally sizes)—to observe if there was underdosage parallel to the direction of the beam (*z* axis) or perpendicular to the beam (*x* or *y* axis). The tally size was set to 1 mm in the direction of interest, and the dimensions of the voxel in the other directions were greater in order to improve statistical results. The step length used in the GAMOS simulations was the default production cut value, and in the Penelope case the values used were EABS(ph) 10 KeV, EABS(e-) 100 KeV and EABS(e+) 100 KeV, according to previously published data [5]. The simulations performed took into account the electron transport.

## RESULTS

**Fig. 4.**
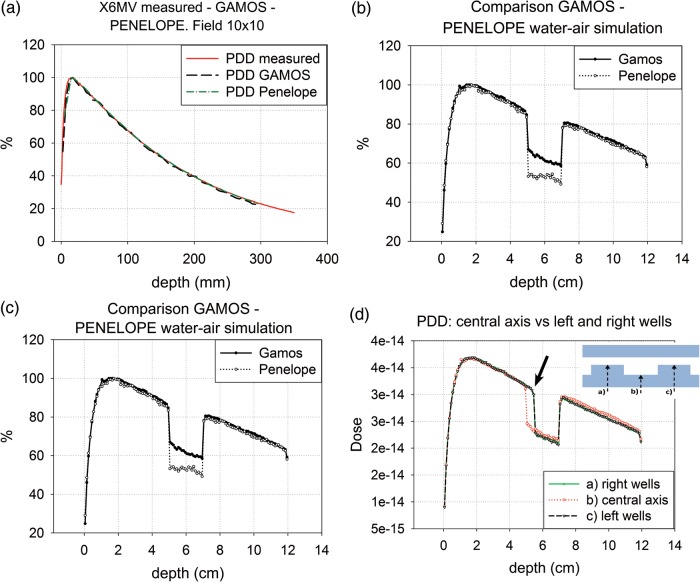
Percentage depth dose (PDD) plots. (**a**) Comparison of measured PDD vs Monte Carlo (MC)-simulated PDD with GAMOS vs MC-simulated PDD with PENELOPE. Field size 10 × 10 cm^2^, SSD = 100 cm, Siemens PRIMUS X6MV. **(b**) Comparison of MC-simulated PPD with Penelope for an homogenous water phantom (30 × 30 × 12 cm^3^) and the experimental configuration [(30 × 30 × (5 water + 2 air + 5 water) cm^3^]. Field size 10 × 10 cm^2^, SSD = 100 cm, Siemens PRIMUS X6MV. (**c**) Comparison of MC-simulated PDD with GAMOS vs MC-simulated PDD with Penelope, with respect to the experimental configuration PDD (5 water + 2 air + 5 water). Different air-density values between the MC codes lead to significant dose differences in the air slab, but these are irrelevant to the goals of this study. Field size 10 × 10 cm^2^, SSD = 100 cm, Siemens PRIMUS X6MV. (**d**) Three MC-simulated PDDs of the culture plate irradiation geometry (Fig. 3) with GAMOS. The two symmetrical plots represent the PDDs of two adjacent central well axes, and the remaining plot represents the PDD crossing through the air midway between the wells, which coincides with the central axis of the irradiation field; this relationship, relative to the culture plate irradiation geometry, is shown in the attached scheme. This plot (4d) was used to obtain the infradosage value in the parallel direction to the radiation field axis (the arrow indicates the voxel chosen to evaluate the infradosage). Simulated plots with Penelope (not shown) were similar. Field size 10 × 10 cm^2^, SSD = 100 cm, Siemens PRIMUS X6MV. Dose in arbitrary units.

**Fig. 5.**
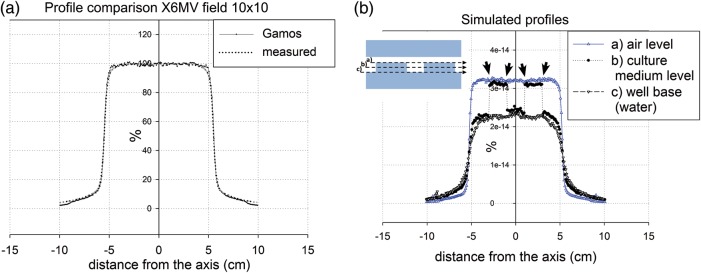
Dose profiles. (**a**) Comparison of measured dose profile vs Monte Carlo (MC)-simulated dose profile with GAMOS. Only subtle differences were observed in the shoulders of the profile, attributable to differences between the IAEA Siemens PRIMUS X6MV spectrum and the real spectrum delivered by our Siemens PRIMUS X6MV LINAC. Field size 10 × 10 cm^2^, depth 10 cm, SSD = 100 cm, Siemens PRIMUS X6MV. (**b**) Simulated dose profiles of the culture plate irradiation geometry with GAMOS at three different depths: at the well base ‘through water’, at the top culture medium level, and at the air level over the wells. This relationship, relative to the culture plate irradiation geometry, is shown in the attached scheme. No relevant lateral underdosage was observed (arrows). Other than that, the penumbras are broadened out in the profiles in contact with air, as expected. The air profile is noisier due to an increase in statistical uncertainty. Field size 10 × 10 cm^2^, SSD = 100 cm, Siemens PRIMUS X6MV.

First, in the homogenous phantom, good agreement between simulated and measured PDDs and profiles was found, both for the 10 × 10 cm^2^ field and the 20 × 20 cm^2^ field (data not shown), and also between the GAMOS and PENELOPE simulation (Fig. 4a). However, a slight difference was observed in the shoulder area of the profile (better appreciated in the profile at the maximum dose depth (Fig. 5a). These divergences probably reflect slight differences in the energy spectrum between the data of phase-spaces proportioned by IAEA and the measured data in our linear accelerator Siemens PRIMUS (Fig. 5a).

According to the simulation in the water–air–water geometry without cell-plates, the underdosage in the last voxel of water adjacent to the water–air interface was 0.955 ± 0.02. However, the measured value was 0.983. Taking into account the fact that the effective point of measurement of the Roos chamber is located at 1 mm from the surface, we corrected the results to the last and penultimate voxel before reaching the water–air interface. A value of 0.984 ± 0.02 was obtained, which tallies very well with the measured data. These prior comparisons between measurements and simulations, therefore, serve to validate the code. Figure 4b shows the comparison between the PDD of the water–air–water phantom with the PDD of the 12-cm homogenous water phantom that was used to estimate the underdosage in this situation. In addition, for this geometry, the comparison between the simulations performed with GAMOS and PENELOPE is included (Fig. 4c). The comparison reveals good agreement except in the air, which is attributable to air density differences between the MC codes, but this result is not of concern for our study.

Finally, after evaluating the results of the plate radiation simulations, we found that the underdosage parallel to the beam direction in the last voxel was 0.965 ± 0.01 (mean of both wells) (Fig. 4d). Perpendicular to the beam direction, we found a negligible lateral underdosage, 0.994 ± 0.01 (average of the two extremes of the two wells) (Fig. 5b). MC calculations of the same plate geometry were repeated with the PENELOPE code and similar results were obtained (data not shown).

## DISCUSSION

No concerns about underdosage as a result of build-down during *in vitro* culture plate irradiaton have been raised by these results. There is little data about the real dosage received inside well culture plates, despite their widespread utilization in radiotherapy research, other than ascertaining that the intended dose is the dose actually delivered. Kulmala *et al*. [6] found that, for 96-well plates, there was a 3% discrepancy between the dosimetric results calculated with a CT-based radiotherapy planning system and those experimentally obtained after dropping thermoluminescent dosimeters (TLDs) into the wells. Tomic *et al.* [7] investigated dose deposition using a radiochromic film and found that the maximum dose gradient was of the order of 1.5% for a 6-MV photon beam. However, analysis of these results needs to take into account the fact that the uncertainties in the measured dose, with the model of film used, were of the order of 2%. Blockhuys *et al*. [8] also used a radiochromic film, but his aim was to investigate the dosimetry related to a ‘smooth linear dose gradient’ using an intensity-modulated beam. Altman *et al*. [9] characterized a phantom for *in vitro* cell experiments comparing TLD and film measurements with those acquired from their treatment planning system. He found differences ranging from 1.3–2.9%, without statistical significance. None of the previous works, however, have dealt with the air–water interface underdosage, thus no direct comparisons can be made with regards to our paper, which is the first to focus on this issue in relation to *in vitro* experiments. TLD doses are an averaged measurement that do not consider spatial dose distribution; radiochromic films have limitations inherent to their nature, such as non-uniformity (which has been reported as ±3% in a multi-institutional investigation), the need for calibration, and the influence related to the scanning system, among others [10, 11].

In our study, we did not evaluate the effect of the energy of the photon beams on the underdosage at the proximate interface. However, the results from Klein *et al*. [12] (using a parallel-plane chamber) indicate a greater underdosage for low-energy photons compared with high-energy photons, owing to a major contribution from back-scattering to the total dose. According to these results, it is advisable to use a photon energy of X18MV (instead of X6MV, which we normally use). The same author also indicated that the thickness of the air gap posterior to the proximate interface scarcely influences the underdosage.

On the other hand, according to Li and coworkers [13], the underdosage at the proximate interface is less dependent on the field size than on the far interface. This can be verified from our graphs (Fig. 4b, c and d), despite this not being the main objective of our study. Although our results relate to a field of 10 × 10 cm^2^, in the light of Li *et al.*'s work, the underdosage obtained at the proximate interface for any other field size is not expected to differ significantly, contrary to what was previously believed.

The differences found between the MC simulations and the measurements are minimal, as revealed by the PDDs and profile plots (Figs 4a and 5a). Despite the fact that the published IAEA phase-spaces have been validated with real measurements, there are some slight differences between IAEA energy spectrum considered to produce the IAEA files used, and the real energy spectrum of our LINAC, but these differences do not affect the results of our study in any significant way.

Comparison of the simulations performed with GAMOS and Penelope indicates good agreement (except in the air, attributable to air density differences between the MC codes, which is of no particular relevance to this study).

## CONCLUSION

In conclusion, we would like to emphasize our finding that in the irradiation of cell culture plates, there is a slight underdosage (∼4%) which corresponds to the last voxel (1 millimeter depth), at the most superficial part of the culture medium, whereas in lower layers where the cells are attached, the underdosage is negligible. The value shown (∼4%) represents the maximum underdosage value found in the whole configuration set-up. We conclude that the underdosage will hardly affect the monolayer of cells at the base of the culture wells. Furthermore, the lateral underdosage is also insignificant.

## FUNDING

This work was partially supported by Research Grant AN-2010/16 from Fundacion Sociosanitaria de Castilla-La Mancha (FISCAM). Funding to pay the Open Access publication charges for this article were provided by Junta local de Albacete de la Asociación Española Contra el Cáncer (AECC).
